# SARS-CoV-2 Cumulative Infection Over the Pandemic and Its Associated Factors Among Healthcare Workers in Japan

**DOI:** 10.2188/jea.JE20250007

**Published:** 2026-03-05

**Authors:** Zobida Islam, Yunfei Li, Shohei Yamamoto, Norio Ohmagari, Naho Morisaki, Makiko Sampei, Koushi Yamaguchi, Kazuyoshi Takeda, Yohei Sasaki, Ryo Okubo, Akihiko Nishikimi, Takeshi Nakagawa, Haruhiko Tokuda, Kunihiro Nishimura, Soshiro Ogata, Kanako Teramoto, Manami Inoue, Takahiro Mita, Mayo Hirabayashi, Maki Konishi, Kengo Miyo, Tetsuya Mizoue

**Affiliations:** 1 Department of Epidemiology and Prevention, Center for Clinical Sciences, Japan Institute for Health Security, Tokyo, Japan; 2 Disease Control and Prevention Center, Japan Institute for Health Security, Tokyo, Japan; 3 Department of Social Science, National Research Institute for Child Health and Development, Tokyo, Japan; 4 Department of Nursing and Social Epidemiology, Nippon Sport Science University, Tokyo, Japan; 5 Center of Maternal-Fetal, Neonatal and Reproductive Medicine, National Center for Child Health and Development, Tokyo, Japan; 6 Clinical Research & Education Promotion Division, National Center Hospital, National Center of Neurology and Psychiatry, Tokyo, Japan; 7 Biosafety Division, Research Institute, National Center for Geriatrics and Gerontology, Aichi, Japan; 8 Department of Social Science, Research Institute, National Center for Geriatrics and Gerontology, Aichi, Japan; 9 Department of Human Sciences, the University of Osaka, Osaka, Japan; 10 Bioresource Division, Research Institute, National Center for Geriatrics and Gerontology, Aichi, Japan; 11 Department of Biostatistics, National Cerebral and Cardiovascular Center, Osaka, Japan; 12 Division of Prevention, Institute for Cancer Control, National Cancer Center, Tokyo, Japan; 13 Division of Cohort Research, Institute for Cancer Control, National Cancer Center, Tokyo, Japan; 14 Center for Medical Informatics Intelligence, Japan Institute for Health Security, Tokyo, Japan

**Keywords:** SARS-CoV-2 infection, seroprevalence, risk factors, healthcare workers, multicenter study

## Abstract

**Introduction:**

Evidence is scarce on cumulative severe acute respiratory syndrome coronavirus 2 (SARS-CoV-2) infections among healthcare workers during the pandemic. This study aimed to describe cumulative infections, including undiagnosed cases, and identify factors associated with infection in healthcare workers in Japan.

**Methods:**

Using serosurveys conducted across six national centers in Japan, we tracked coronavirus disease 2019 (COVID-19) cumulative infections. Seropositivity was defined as a positive result for SARS-CoV-2 nucleocapsid protein using the Roche assay, and cumulative infection was defined as the proportion of participants who tested positive for anti-nucleocapsid antibodies and/or self-reported a history of laboratory-confirmed or clinically diagnosed COVID-19 since the start of the pandemic. A robust Poisson regression model was used to investigate factors associated with infection risk as of September 2023.

**Results:**

Cumulative infection, which was less than 5% until the end of 2021, increased after the emergence of the Omicron variant. Specifically, cumulative infection reached 14.6% in July 2022 (BA.1/2), 37.4% in December 2022 (BA.5), 53.3% in September 2023 (XBB subvariants), and 71.5% in December 2023 (JN.1 subvariant). The proportion of undiagnosed cases detected by antibody testing alone, without a prior diagnosis, decreased from 60.9% in December 2020 to 24.7% in December 2023. Individuals aged 50 to 59 years (prevalence ratio [PR] 0.73; 95% confidence interval [CI], 0.67–0.79) and 60 years or older (PR 0.67; 95% CI, 0.59–0.77) had lower cumulative infections than those aged under 30 years old. Physicians and nurses had significantly higher cumulative infections than administrative staff, with fully adjusted PR of 1.09 (95% CI, 1.01–1.18) and 1.18 (95% CI, 1.08–1.30), respectively.

**Conclusion:**

Among healthcare workers in Japan, cumulative SARS-CoV-2 infection markedly increased after the emergence of the Omicron variant, whereas the proportion of undiagnosed cases has decreased throughout the pandemic. Younger people (<50 years), as well as physicians and nurses, have faced a higher risk of infection.

## INTRODUCTION

The epidemic of coronavirus disease 19 (COVID-19) caused by severe acute respiratory syndrome coronavirus 2 (SARS-CoV-2) has continued for more than 4 years.^1^^,^^2^ Since the emergence of the Omicron variant, the cumulative number of confirmed cases has sharply increased from 82.9 million in December 2020 (approximately 1.1% of the global population) to 767 million by June 2023 (nearly 10% of the global population).^3^ Most infections were mild or asymptomatic (91.4% during Delta and 97.9% during Omicron waves),^4^ which may lead to a lower chance of receiving diagnostic tests and produce a large number of undiagnosed cases.^5^ In this regard, serosurveys are a valuable tool for estimating the total cases of infection, including undiagnosed ones.^6^

Healthcare workers are a group at high risk of contracting COVID-19. A meta-analysis conducted in the early phase of the pandemic (as of December 2020) reported a higher seroprevalence of SARS-CoV-2 antibodies among high-risk healthcare workers than that among the general population (17.1% versus 8.0%).^7^ During the Omicron-predominant period, cross-sectional studies of healthcare workers in Japan^8^ and Switzerland^9^ using seroprevalence data reported high proportions (22.8–43.9%) of SARS-CoV-2 infection. These studies also found that many healthcare workers were unaware of their infections.^8^^–^^10^ Repeated serosurveys among this occupational group can help to capture the spread of the infection, including those undiagnosed, which may trigger outbreaks among healthcare workers and patients.

Data on the trend of cumulative SARS-CoV-2 infections—the proportion of individuals ever infected since the start of the pandemic, including undiagnosed cases—over the pandemic are lacking among healthcare workers. In Japan, one study among healthcare workers of a university hospital in Tokyo reported repeatedly measured seroprevalence, rising from 2.8% in April 2020 to 54.1% in June 2023.^11^ However, this study^11^ did not encompass all SARS-CoV-2 variants and subvariants. Given the rapid spread of the Omicron variants and their subvariants across Japan since 2022, more comprehensive studies with updated data on seroprevalence are needed.

Studies among healthcare workers during the early period of the pandemic identified working in an intensive care unit and having close contact with COVID-19 patients as risk factors for SARS-CoV-2 infection^12^^–^^15^; however, later studies in hospitals with good infection control measures demonstrated that these occupational factors were not associated with SARS-CoV-2 infection risk.^16^^–^^18^ Among studies conducted in the late stage of the pandemic, only three studies investigated the occupational risk among healthcare workers during the Omicron waves.^17^^,^^19^^,^^20^

The present study aimed to 1) describe the trends in cumulative SARS-CoV-2 infections, including undiagnosed cases, over the pandemic, including major Omicron waves, and 2) clarify background factors associated with the infection during the Omicron epidemic period, using data from repeated serosurveys among workers at six national centers for advanced medical research in Japan.

## METHODS

### Study setting and survey

We launched a multicenter collaborative serological study on COVID-19 targeting healthcare workers at six National Centers for Advanced Medical and Research (NCs) in Japan; namely, National Cancer Center (NCC), National Center for Child Health and Development (NCCHD), National Center for Geriatrics and Gerontology (NCGG), Japan Institute for Health Security (JIHS) (formerly National Center for Global Health and Medicine [NCGM]), National Center of Neurology and Psychiatry (NCNP), and National Cerebral and Cardiovascular Center (NCVC). The researchers of the six NCs agreed on the basic protocol of the survey, including schedule, questionnaire, and antibody testing.^21^ Each NC performed the survey at least once per year during the COVID-19 epidemic and provided the data to the steering committee (JIHS) for pooling purposes. The participation rate varied across these centers and survey years, depending on the target of each survey (eTable 1). Written and electronic informed consent was obtained from each participant. After completing the opt-out process, the survey data were anonymized and submitted to the study committee for pooled analysis. The study design and procedure for data collection were approved by the ethical committee of each center, while that of the pooling study was approved by the ethics committee of the JIHS (approved number: NCGM-G-004233).

### Study design and participants

**Figure 1.**
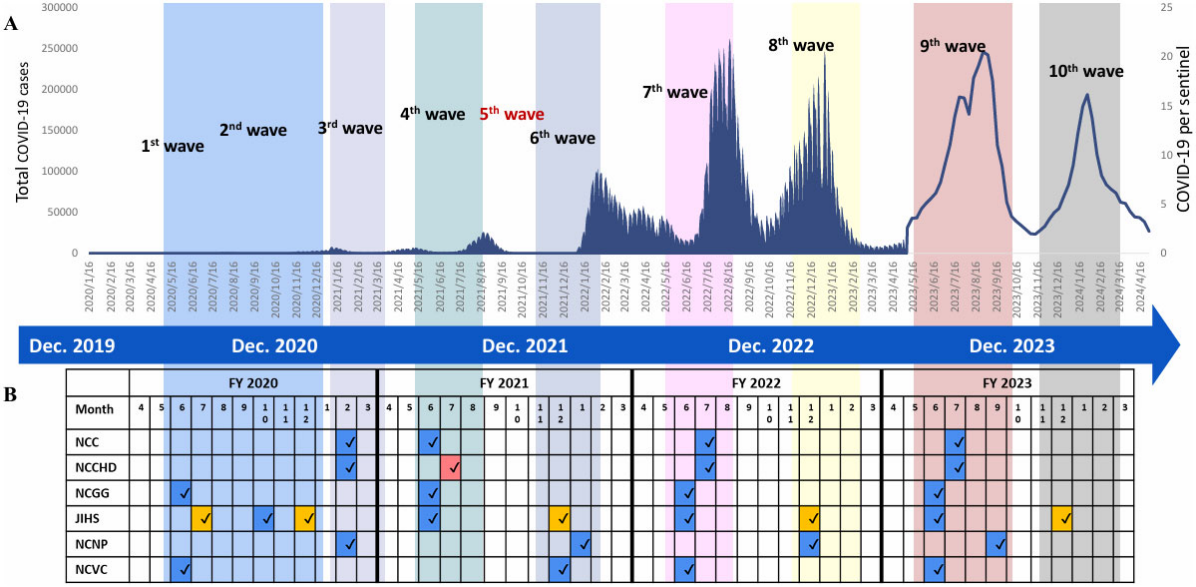
Change in the number of newly confirmed COVID-19 cases in Japan (**A**): until April 2023, the data represent all reported COVID-19 cases nationwide; from May 2023 onward, the data represent the weekly number of reported cases per sentinel, based on Japan’s national sentinel surveillance system, which monitors infectious disease trends through a network of selected medical institutions. (**B**) shows the timeline of antibody surveys among workers of the 6 national centers during the pandemic (orange colored cell indicates the timeline for additional surveys). ^*^Blue-colored cells indicate the availability of N antibody data from NCs according to the survey timing. Yellow-colored cells represent the availability of N antibody data from additional sero-surveys. Red-colored cell indicates that this center was not included in the period 3 analysis, as it lacked data on N antibodies. ^Ψ^It was not possible to define any time period for the 5th wave because no serosurvey was conducted in relation to it. JIHS, Japan Institute for Health Security; NCC, National Cancer Center; NCCHD, National Center for Child Health and Development; NCGG, National Center for Geriatrics and Gerontology; NCNP, National Center of Neurology and Psychiatry; NCVC, National Cerebral and Cardiovascular Center.

The present study is a repeated cross-sectional study using pooled data. We grouped serosurveys into eight time periods according to the survey timing in relation to the COVID-19 epidemic waves in Japan (Figure 1). Specifically, time period 1 was between July and December 2020 including data from 3 centers (NCGG, JIHS, and NCVC); time period 2 was February 2021 including data from 3 centers (NCC, NCCHD, and NCNP); time period 3 was July 2021 including data from 3 centers (NCC, NCGG, and JIHS); time period 4 was between December 2021 and January 2022 including data from 3 centers (JIHS, NCNP, and NCVC); time period 5 was between June and July 2022 including data from 5 centers (NCC, NCCHD, NCGG, JIHS, and NCVC); time period 6 was December 2022 including data from 2 centers (JIHS and NCNP); time period 7 was between June and September 2023 including data from all 6 centers; and time period 8 was December 2023 including data from JIHS. The number of participants for time periods 1 through 8 was 3,762, 1,462, 4,170, 2,087, 6,631, 2,046, 6,287, and 1,651, respectively.

Regarding the risk factor analysis, we analyzed the pooled data of 6 NCs in period 7 (June to September 2023). We excluded those with any missing covariates (*n* = 66) on sex, age, job, affiliated department, and occupational risk of SARS-CoV-2 infection, leaving 6,221 participants for analysis.

### Detection of SARS-CoV-2 infection

The main outcome was cumulative SARS-CoV-2 infection, which was defined as the proportion of participants who tested positive for anti-nucleocapsid antibodies (indicative of past natural infection) and/or self-reported a history of COVID-19 since the start of the pandemic. The mRNA-based COVID-19 vaccines, which are the main type used in Japan, do not encode the SARS-CoV-2 nucleocapsid protein and do not induce anti-nucleocapsid antibodies. We qualitatively measured total antibodies, including IgG, against the SARS-CoV-2 nucleocapsid protein (Roche Elecsys^®^ Anti-SARS-CoV-2; Roche, Basel, Switzerland). The sensitivity of the Roche assay is persistently high, even for more than 1 year: that on 60 and 430 days after infection was reported as 99.5% and 97.1%, respectively.^22^ A seropositive case was defined as a positive result on the Roche assay (≥1.0 cutoff index). A history of COVID-19 included both (1) laboratory-confirmed cases (diagnosed by a physician based on a polymerase chain reaction (PCR) or antigen test) and (2) clinically-diagnosed cases without laboratory testing (symptoms compatible with COVID-19 following close contact with a patient with COVID-19). In JIHS, the self-report of COVID-19 was verified against the in-house registry. Undiagnosed cases were defined as the undiagnosed SARS-CoV-2 infection detected with an antibody test only. The proportion of undiagnosed cases was calculated by the formula below:

$$\begin{array}{rl} & \text{Proportion of undiagnosed cases}\\ & \;=1-\frac{\text{Diagnosed cases}}{\text{Total cases}\;(\text{diagnosed cases}+\text{undiagnosed cases})} \end{array}$$ (1)

### Background factors

Factors include demographic characteristics (sex and age), occupational factors (job category), and the location of each center (Tokyo or outside Tokyo), as these factors were shown to be associated with SARS-CoV-2 infection.^23^ Age was classified into five groups: <30, 30–39, 40–49, 50–59, or ≥60 years. Occupation was classified into six groups: doctor, nurse, allied health professionals, administrative staff, researcher, or others. As for the location of NCs, we classified centers as Tokyo (NCC, NCCHD, NCNP, and JIHS) or non-Tokyo (NCGG and NCVC).

Because the degree of potential occupational exposure to SARS-CoV-2 and vaccination status have changed throughout the pandemic, and the timing of infections in relation to these events could not be determined, these factors were not included in the analysis of associations. They were only included to describe the background characteristics of the participants. Possible occupational exposure to SARS-CoV-2 was assessed using the following two self-reported questions: “Have you ever engaged in COVID-19-related work since January 2023?” and “Did you engage in any work in which you were heavily exposed to SARS-CoV-2 since January 2023?”. Participants were categorized into three groups as follows: low (not engaged in COVID-19-related work), moderate (engaged in COVID-19-related work without heavy exposure to SARS-CoV-2), or high (heavily exposed to SARS-CoV-2). The vaccination status was classified into four groups: unvaccinated, 1–2 doses, 3 doses, ≥4 doses, or unknown.

### Statistical analysis

We calculated the following infection indicators for each period of the pandemic: the number and proportion of diagnosed cases, seropositive cases, and cases with a prior SARS-CoV-2 infection. We also calculated the proportion of undiagnosed cases among the total cases. In association analysis, we estimated the prevalence ratio (PR) with a 95% confidence interval (CI) using robust Poisson regression models. Model 1 was adjusted for sex and age. Model 2 was additionally adjusted for job and location of NCs. We also performed a sensitivity analysis categorizing NCs into three prefectural groups (Tokyo, Osaka, or Aichi) to account for regional differences in prevalence. All statistical analyses were performed with the Stata 18.0 software (Stata Corp LLP, College Station, TX, USA).

## RESULTS

As shown in Table 1, among participants of the fourth survey (June to September 2023), 73.1% of them were women, and 68.9% worked in Tokyo; major occupations were nurses (34.2%), allied healthcare professionals (15.4%), physicians (14.6%), administrative staff, and researchers (15.3%). A total of 14.5% worked in a COVID-19-affiliated department, and 38.0% had a moderate or high risk of occupational exposure to SARS-CoV-2. As regards vaccination, approximately 90% of the participants in the survey of July 2021 completed the second dose before the survey, and more than 70% of the participants in the survey of July 2022 completed the third dose. Approximately 70% of the participants in the June to September 2023 survey completed the fourth dose of vaccine before the period of the survey.

**Table 1.** Characteristics of the study participants in each time point during the COVID-19 pandemic

	Survey timing							

	Dec. 2020	Feb. 2021	Jul. 2021	Jan. 2022	Jul. 2022	Dec. 2022	Sep. 2023	Dec. 2023
**Total**	3,762	1,462	4,170	2,087	6,631	2,046	6,287	1,651
**Location of the centers, *N* (%)**								
Tokyo	2,563 (68.1)	1,462 (100)	3,370 (80.8)	1,355 (64.9)	4,619 (69.7)	2,046 (100)	4,649 (74.0)	1,651 (100)
Outside Tokyo	1,199 (31.9)	0	800 (19.2)	732 (35.1)	2,012 (30.3)	0	1,638 (26.0)	0
**Sex, *N* (%)**								
Men	1,147 (30.4)	387 (26.5)	1,209 (29.0)	574 (27.5)	1,804 (27.2)	547 (26.7)	1,650 (26.2)	458 (27.7)
Women	2,581 (68.6)	1,074 (73.4)	2,936 (70.4)	1,406 (67.4)	4,789 (72.2)	1,494 (73.0)	4,591 (73.1)	1,192 (72.2)
Missing	34 (1.0)	1 (0.1)	25 (0.6)	107 (5.1)	38 (0.6)	5 (0.3)	46 (0.7)	1 (0.1)
**Age, *N* (%)**								
<30 years	1,044 (27.7)	252 (17.2)	1,137 (27.3)	701 (33.6)	1,828 (27.6)	539 (26.3)	1,605 (25.5)	506 (30.6)
30–39 years	925 (24.6)	380 (26.0)	1,034 (24.8)	560 (26.8)	1,683 (25.4)	473 (23.1)	1,468 (23.4)	366 (22.2)
40–49 years	943 (25.1)	460 (31.5)	1,054 (25.2)	416 (19.9)	1,581 (23.8)	517 (25.3)	1,561 (24.8)	358 (21.7)
50–59 years	613 (16.3)	293 (20.0)	693 (16.6)	243 (11.6)	1,135 (17.1)	374 (18.3)	1,192 (19.1)	281 (17.0)
≥60 years	198 (5.3)	75 (5.1)	220 (5.3)	59 (2.8)	360 (5.4)	140 (6.8)	425 (6.8)	138 (8.4)
Missing	39 (1.0)	2 (0.1)	32 (0.8)	108 (5.2)	44 (0.7)	3 (0.2)	0 (0.0)	2 (0.1)
**Occupation, *N* (%)**								
Physician	523 (13.9)	174 (11.9)	572 (13.7)	253 (12.1)	973 (14.7)	282 (13.8)	916 (14.6)	238 (14.4)
Nurse	1,343 (35.7)	467 (31.9)	1,403 (33.6)	1,004 (48.1)	2,406 (36.3)	728 (35.5)	2,148 (34.2)	611 (37.0)
Allied healthcare professional	674 (17.9)	230 (15.7)	694 (16.6)	311 (14.9)	1,045 (15.8)	262 (12.8)	970 (15.4)	249 (15.1)
Administrative staff	518 (13.8)	256 (17.5)	582 (14.0)	139 (6.7)	892 (13.4)	351 (17.2)	964 (15.3)	242 (14.7)
Researcher	473 (12.6)	299 (20.4)	675 (16.2)	229 (11.0)	946 (14.3)	324 (15.8)	964 (15.3)	238 (14.4)
Other	185 (4.9)	35 (2.4)	203 (4.9)	36 (1.7)	301 (4.5)	96 (4.7)	278 (4.4)	71 (4.3)
Missing	46 (1.2)	1 (0.1)	41 (1.0)	115 (5.5)	68 (1.0)	3 (0.2)	47 (0.8)	2 (0.1)
**Degree of possible occupational exposure to SARS-CoV-2, *N* (%)**								
Low	1,777 (47.2)	934 (63.9)	2,349 (56.3)	1,000 (47.9)	3,905 (58.9)	1,135 (55.5)	3,855 (61.3)	1,010 (61.2)
Moderate	1,251 (33.2)	363 (24.8)	1,160 (27.8)	537 (25.7)	1,601 (24.1)	537 (26.3)	1,371 (21.8)	362 (21.9)
High	688 (18.3)	164 (11.2)	611 (14.6)	443 (21.2)	1,071 (16.1)	371 (18.1)	1,020 (16.2)	277 (16.8)
Missing	46 (1.2)	1 (0.1)	50 (1.2)	107 (5.1)	54 (0.8)	3 (0.2)	41 (0.7)	2 (0.1)
**Vaccination, *N* (%)**								
Unvaccinated	—	—	274 (6.6)	42 (2.0)	118 (1.8)	37 (1.8)	116 (1.9)	20 (1.2)
1–2 doses	—	—	3,721 (89.2)	1,266 (60.7)	307 (4.6)	82 (4.0)	248 (3.9)	48 (2.9)
3 doses	—	—	—	315 (15.1)	5,850 (88.2)	588 (28.7)	1,351 (21.5)	392 (23.7)
≥4 doses	—	—	—	—	146 (2.2)	1,312 (64.1)	4,388 (69.8)	1,149 (69.6)
Missing	—	—	175 (4.2)	464 (22.2)	210 (3.2)	27 (1.3)	184 (2.9)	42 (2.5)

COVID-19, coronavirus disease 2019; SARS-CoV-2, severe acute respiratory syndrome coronavirus 2.

^*^The vaccination status of NCVC and NCNP was not available in the surveys between December 2021 and January 2022. Regarding the location of the centers, Tokyo includes 4 centers (National Cancer Center, National Center for Child Health and Development, National Center of Neurology and Psychiatry, and Japan Institute for Health Security); outside Tokyo includes 2 centers (National Center for Geriatrics and Gerontology and National Cerebral and Cardiovascular Center).

**Figure 2.**
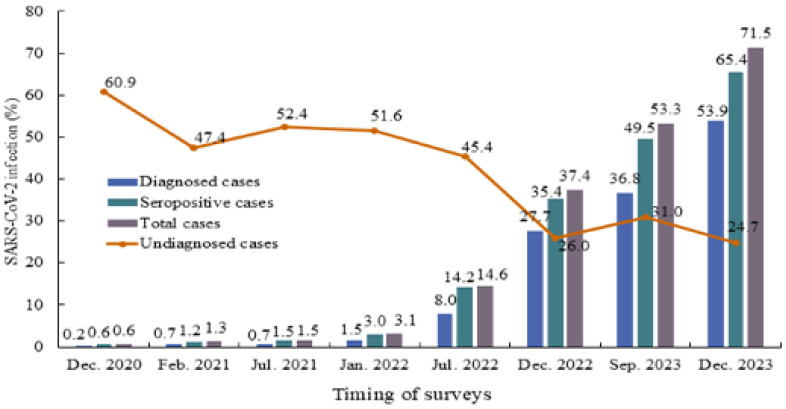
Trends in SARS-CoV-2 cumulative infection (%) and undiagnosed cases (%) among workers of the 6 national centers in Japan. SARS-CoV-2, severe acute respiratory syndrome coronavirus 2.

Cumulative infection was low before July 2021 (1.5% as of July 2021). The proportion of cumulative infection slightly increased from 1.5% in the survey of July 2021 to 3.1% in January 2022; between these surveys, the Delta-predominant epidemic occurred during the summer season (Table 2 and Figure 2). The cumulative infection showed a rapid increase after the emergence of Omicron variants; it increased to 14.6% at surveys in July 2022 after the Omicron BA.1/2 predominant epidemic, 37.4% at survey in December 2022 after the Omicron BA.5 predominant epidemic, and 53.3% at surveys between June and September 2023 after the Omicron XBB variants epidemic, 71.5% at surveys in December after the Omicron JN.1 subvariant epidemic (Table 2 and Figure 2).

**Table 2.** Trends in infection indicators during the COVID-19 pandemic among workers of 6 national centers

	Survey timing							

	Dec. 2020	Feb. 2021	Jul. 2021	Jan. 2022	Jul. 2022	Dec. 2022	Sep. 2023	Dec. 2023
**Participants, *N***	3,762	1,462	4,170	2,087	6,631	2,046	6,287	1,651
**Diagnosed case, *N* (%)**	9 (0.2)	10 (0.7)	30 (0.7)	31 (1.5)	527 (8.0)	567 (27.7)	2,312 (36.8)	889 (53.9)
**Seropositive case, *N* (%)**	21 (0.6)	18 (1.2)	62 (1.5)	62 (3.0)	943 (14.2)	724 (35.4)	3,114 (49.5)	1,080 (65.4)
**Total case, *N* (%)**	23 (0.6)	19 (1.3)	63 (1.5)	64 (3.1)	965 (14.6)	766 (37.4)	3,353 (53.3)	1,180 (71.5)
**Undiagnosed case^a^, %**	60.9	47.4	52.4	51.6	45.4	26.0	31.0	24.7
**Ratio of total cases to diagnosed cases**	2.5	1.9	2.1	2.1	1.8	1.4	1.5	1.3

^a^Proportion of undiagnosed cases (seropositive only) among total cases.

The proportion of undiagnosed cases among total infections showed a decreasing trend over time, from 60.9% in December 2020 to 31.0% in the period between June and September 2023. There was a temporal increase (52.4%) in July 2021 due to the Delta variant, followed by a downward trend during the epidemic of the Omicron variant. Subgroup analyses by sex, age category, job category, and location of NCs showed similar trends (eFigure 1, eFigure 2, eFigure 3, and eFigure 4). Participants aged 50 years or older showed a lower proportion of cumulative infection than those aged under 50 years (eFigure 2).

Table 3 shows the association between background factors and cumulative SARS-CoV-2 infections as of September 2023. In a fully adjusted model (model 2), people aged 50 to 59 years (PR 0.73; 95% CI, 0.67–0.79) and 60 years or older (PR 0.67; 95% CI, 0.59–0.77) had lower cumulative infections than those aged under 30 years old. Physicians (PR 1.18; 95% CI, 1.08–1.30) and nurses (PR 1.09; 95% CI, 1.01–1.18) had a slightly higher risk of cumulative infection than administrative staff. These results remained virtually unchanged when NCs were categorized based on their prefectural locations (Tokyo, Osaka, or Aichi) (eTable 2).

**Table 3.** Robust Poisson regression for the association between background factors and SARS-CoV-2 infection among the workers of 6 national centers in June/July/September 2023

	Number of participants	Cumulative infection, *n* (%)	Undiagnosed case, *n* (%)	PR (95% CI)	

				Model 1	Model 2
**Total**	6,221	3,317 (53.3)	1,015 (30.6)		
**Sex**					
Men	1,641	882 (53.7)	304 (34.5)	Ref.	Ref.
Women	4,580	2,435 (53.2)	711 (29.2)	0.97 (0.92–1.02)	0.99 (0.94–1.05)
**Age, years**					
<30	1,597	929 (58.2)	307 (33.0)	Ref.	Ref.
30–39	1,452	853 (58.8)	239 (28.0)	1.01 (0.95–1.07)	1.01 (0.95–1.08)
40–49	1,557	878 (56.4)	253 (28.8)	0.97 (0.91–1.03)	0.98 (0.92–1.04)
50–59	1,190	494 (41.5)	151 (30.6)	**0.71 (0.66–0.77)**	**0.73 (0.67–0.79)**
≥60	425	163 (38.4)	65 (39.9)	**0.66 (0.58–0.74)**	**0.67 (0.59–0.77)**
**Occupation**					
Physician	910	551 (60.6)	181 (32.81)	**1.18 (1.08–1.30)**	**1.18 (1.08–1.30)**
Nurse	2,141	1,217 (56.8)	357 (29.3)	**1.09 (1.01–1.18)**	**1.09 (1.01–1.18)**
Allied health professional	968	472 (48.8)	111 (23.5)	0.96 (0.87–1.05)	0.96 (0.87–1.05)
Administrative staff	964	451 (46.8)	135 (27.7)	Ref.	Ref.
Researcher	962	495 (51.5)	178 (36.0)	1.06 (0.97–1.16)	1.06 (0.97–1.16)
Other	276	131 (47.5)	53 (40.4)	1.04 (0.91–1.20)	1.04 (0.91–1.20)
**Location of the center**					
Outside Tokyo	1,587	835 (52.6)	287 (34.4)	Ref.	Ref.
Tokyo	4,634	2,482 (53.6)	728 (29.3)	1.03 (0.98–1.09)	1.03 (0.97–1.08)

CI, confidence interval; PR, prevalence ratio; SARS-CoV-2, severe acute respiratory syndrome coronavirus 2.

Model 1 was adjusted for age and sex. Model 2 was additionally adjusted for job (physician, nurse, allied health professional, administrative staff, researcher, or others) and location of NCs (Tokyo or outside Tokyo).

Regarding the location of the centers, Tokyo includes 4 centers (National Cancer Center, National Center for Child Health and Development, National Center of Neurology and Psychiatry, and Japan Institute for Health Security); outside Tokyo includes 2 centers (National Center for Geriatrics and Gerontology and National Cerebral and Cardiovascular Center).

## DISCUSSION

Using data from repeated serosurveys of healthcare workers at national medical research institutes in Japan, we confirmed a very low SARS-CoV-2 seroprevalence in the early phase of the pandemic, followed by a sharp increase after the emergence of the Omicron variants. By December 2023, it is estimated that more than 70% of workers were infected with SARS-CoV-2. By September 2023, nearly one-third of infections were undiagnosed, though this proportion has decreased throughout the pandemic. A higher infection risk was observed among younger workers under 50 years old, as well as physicians and nurses.

### Low seroprevalence in the early period

The low seroprevalence observed in our study (<1% as of December 2020) is consistent with findings from neighboring countries, such as South Korea^24^^,^^25^ and Taiwan,^26^^,^^27^ where healthcare workers also exhibited seroprevalence below 1% during the early phase of the pandemic. It is worth noting that Japan experienced a much lower infection rate among healthcare workers during this period compared to many other countries. Specifically, the seroprevalence in our study is less than 1% as of December 2020, which was much lower than the seroprevalence among healthcare workers (8% as of December 2020), estimated in a systematic review of studies in China, the United States, India, and some European countries.^28^ In the early pandemic period (as of May 8, 2020), a high number of deaths among healthcare workers were documented in Italy (*n* = 220), the United States (*n* = 202), and the United Kingdom (*n* = 163), whereas no deaths due to COVID-19 were reported among healthcare workers in Japan.^23^ The much lower infection and mortality in Japanese healthcare workers can be attributed to multiple infection control measures adopted in hospitals since the early phase of the epidemic. These include the provision of training for healthcare workers treating COVID-19 patients, personal protective equipment, universal masking, hand washing, routine checking of workers’ body temperature, PCR testing in case of suspected infection, and periodic advisory e-mail messages to the workers.^17^^,^^29^^,^^30^ The present finding of a very low seroprevalence in the early phase of the pandemic indirectly supports the effectiveness of the infection prevention measures implemented to protect healthcare workers during that period.

### Trend of seroprevalence

Direct comparisons of seroprevalence between studies require caution. However, the seroprevalence in our study since the emergence of the Omicron variant (14.6% in July 2022, 37.4% in December 2022, and 53.3% between June and September 2023) is comparable to that reported among healthcare workers at a university hopital in Tokyo (17.7% in June 2022, and 54.1% in June 2023, measured using the Roche assay).^11^ Besides, the observed seroprevalence in our study was notably higher than those serosurveys, using the Roche assay, conducted among the Japanese general population across five prefectures (4.3% in February 2022,^31^ 25.9% in December 2022, and 31.6% in February 2023^32^) and moderately higher than the seroprevalence observed among blood donors of 47 prefectures (28.6% in November 2022)^33^ and 22 prefectures (45.3% in August 2023).^34^ This higher seroprevalence among healthcare workers may be explained by their greater chance of exposure to SARS-CoV-2 during the care of patients, irrespective of COVID-19 status. Additionally, the highly transmissible nature of the Omicron variant,^35^ along with its large proportion of asymptomatic or mildly symptomatic cases,^4^^,^^10^ may have facilitated the spread of infection in healthcare settings. It is thus essential to maintain preventive measures, such as routine health checks, good hygiene practices, and universal masking in healthcare settings.

### Undiagnosed case trend

The presence of a large number of asymptomatic infections or infections with mild symptoms is an important feature of COVID-19 and explains its widespread infection across the community. In the present study population, the proportion of undiagnosed cases, which was high in the early phase of the pandemic (60.9%), showed a decreasing trend over time, reaching 31.0% in September 2023 and 24.7% in December 2023. This could be due, at least in part, to the improved access to and availability of diagnostic tests for the workers, including a self-testing program using antigen test kits.

Physicians and nurses had a higher risk of SARS-CoV-2 infection compared with other occupations. This finding is consistent with previous studies among healthcare workers in England and Wales^36^ and Italy^37^ during the Omicron period. In our analysis, the association did not materially change after controlling for potential occupational exposure to SARS-CoV-2. One plausible explanation is that physicians and nurses have a greater chance of exposure to the virus through direct and closer contact with patients who were hospitalized due to health problems other than COVID-19, some of whom might have asymptomatic SARS-CoV-2 infection. Additionally, our data showed that the proportion of individuals with a high degree of possible occupational exposure was markedly higher among physicians (37.6%) and nurses (47.6%) than among other occupational groups (0.5% to 2.4%), further supporting this interpretation. More effort should be placed on the protection of healthcare workers against nosocomial infections.

We found that people aged 50 years and older were less likely to be infected than those aged under 50 years. This finding is comparable to the results of a serosurvey study conducted from July through August 2023 among the general population of Japan.^34^ The lower prevalence of infection among older adults may be due to their greater adherence to recommended protective practices against infection (eg, wearing masks, maintaining good hygiene, and practicing social distancing). In our previous 2020 JIHS study,^38^ adherence to infection prevention practices increased with age. The ORs for good adherence were 1.00 (reference), 1.33, 1.63, and 2.53 for those aged <30 years, 30 to 39 years, 40 to 49 years, and ≥50 years, respectively. Taken together, more attention should be directed toward younger healthcare workers during periods when viruses with high transmission potential are circulating widely.

The strengths of the present multicenter study include its large sample size and its repeated antibody measurements spanning the early period of the pandemic through the Omicron variant era. However, we should also acknowledge the study’s limitations. First, information on the history of COVID-19 was obtained via self-report, which is subject to recall bias. Nevertheless, the majority of individuals with a history of COVID-19 were seropositive. For example, 78% of diagnosed cases were seropositive in December 2020, and this proportion increased to 89% by December 2023. Therefore, reporting error is unlikely to strongly influence our findings. Second, participation rates in the current study varied by center and survey year. However, differences in seroprevalence among centers within each fiscal year were small (eTable 3). Thus, the possibility of bias due to selective participation in the study is likely minimal. Third, we used complete-case analysis for the risk factor analysis. However, missing data were few (1.0%), so any potential bias introduced by this exclusion would be negligible. Finally, this study was conducted on individuals employed at national medical research institutions that specialize in specific clinical fields. Thus, the current results may not apply to medical professionals working at general clinics and hospitals.

### Conclusions

Among the workers of 6 national medical research centers in Japan, cumulative SARS-CoV-2 infections (including undiagnosed cases), which were very low in the early period of the pandemic, dramatically increased after the emergence of the Omicron variant. A sizable portion of the infections were undiagnosed, though this proportion decreased over time. Younger healthcare workers aged under 50 years, as well as physicians and nurses, were at a higher risk of SARS-CoV-2 infection than their counterparts during the pandemic.

## SUPPLEMENTARY MATERIAL

eTable 1. Number of participants across the fiscal year survey

eTable 2. Robust Poisson regression for the association between background factors and SARS-CoV-2 infection among the workers of 6 national centers in June/July/September 2023

eTable 3. COVID-19 Seroprevalence of each National Center across the fiscal year survey

eFigure 1. Trends in SARS-CoV-2 infection among staff at the six national centers in Japan during the pandemic, showing the percentage of diagnosed cases (**A**), seropositive cases (**B**), total cases (**C**), and undiagnosed cases (**D**) by sex

eFigure 2. Trends in SARS-CoV-2 infection among staff at the six national centers in Japan during the pandemic, showing the percentage of diagnosed cases (**A**), seropositive cases (**B**), total cases (**C**), and undiagnosed cases (**D**) by age category

eFigure 3. Trends in SARS-CoV-2 infection among staff at the six national centers in Japan during the pandemic, showing the percentage of diagnosed cases (**A**), seropositive cases (**B**), total cases (**C**), and undiagnosed cases (**D**) by job category

eFigure 4. Trends in SARS-CoV-2 infection among staff at the six national centers in Japan during the pandemic, showing the percentage of diagnosed cases (**A**), seropositive cases (**B**), total cases (**C**), and undiagnosed cases (**D**) by location of national centers
