# Supplementary material for: SARS-CoV-2 Cumulative Infection Over the Pandemic and Its Associated Factors Among Healthcare Workers in Japan
Source: J Epidemiol. 2026 Mar 5;36(3):107–14. doi: 10.2188/jea.JE20250007 (PMC12893839; doi:10.2188/jea.JE20250007)
Supplement: Supplementary file 1 [file je-36-107-s001.pdf]

eTable 1. Number of participants across the fiscal year survey

	2020 fiscal year			2021 fiscal year			2022 fiscal year			2023 fiscal year		
**NCs**	**Invited**	**Participated (%)**	**Antibody tested (%)**	**Invited**	**Participated (%)**	**Antibody tested (%)**	**Invited**	**Participated (%)**	**Antibody tested (%)**	**Invited**	**Participated (%)**	**Antibody tested (%)**
**NCC**	2,361	451 (19.0)	444 (19.0)	2,432	613 (25.2)	607 (25.0)	587	513 (87.4)	522 (88.9)	516	413 (80.0)	429 (83.1)
**NCCHD**	2,204	830 (37.7)	361 (16.4)	2,204	1,496 (67.9)	--	1,975	1,402 (71.0)	1,402 (71.0)	1,952	1,308 (67.0)	1,308 (67.0)
**NCGG**	743	609 (82.0)	631 (84.9)	878	711 (81.0)	800 (91.1)	921	763 (82.8)	806 (87.5)	936	542 (57.9)	553 (59.0)
**JIHS**	2,893	2,480 (85.7)	2,563 (88.6)	3,072	2,699 (87.9)	2,763 (89.9)	3,181	2,685 (84.4)	2,695 (84.7)	3,206	2,618 (81.6)	2,596 (80.9)
**NCNP**	1,437	657 (45.7)	657 (45.7)	1,435	407 (28.4)	407 (28.4)	1,400	369 (26.4)	369 (26.4)	1,400	316 (22.5)	316 (22.5)
**NCVC**	1,800	772 (42.9)	568 (31.6)	1,168	297 (25.4)	732 (62.7)	1,871	1,166 (62.3)	1,206 (64.5)	1,797	1,056 (58.7)	1,085 (60.3)
**Total**	11,438	5,799 (50.7)	5,224 (45.7)	11,189	6,223 (55.6)	5,309 (47.4)	9,935	6,898 (69.4)	7,000 (70.5)	9,807	6,253 (63.7)	6,287 (64.1)

JIHS, Japan Institute for Health Security; NC, national center; NCC, National Cancer Center; NCCHD, National Center for Child Health and Development; NCGG, National Center for Geriatrics and Gerontology; NCNP, National Center of Neurology and Psychiatry; NCVC, National Cerebral and Cardiovascular Center.

eTable 2. Robust Poisson regression for the association between background factors and SARS-CoV-2 infection among the workers of 6 national centers in June/July/September 2023

	Number of participants	Cumulative infection, n (%)	PR (95% CI)	
			Model 1	Model 2
**Total**	6,221	3,317 (53.3)		
**Sex**				
Men	1,641	882 (53.7)	Ref.	Ref.
Women	4,580	2,435 (53.2)	0.97 (0.92–1.02)	0.99 (0.93–1.05)
**Age, years**				
<30	1,597	929 (58.2)	Ref.	Ref.
30–39	1,452	853 (58.8)	1.01 (0.95–1.07)	1.01 (0.95–1.08)
40–49	1,557	878 (56.4)	0.97 (0.91–1.03)	0.98 (0.92–1.04)
50–59	1,190	494 (41.5)	**0.71 (0.66–0.77)**	**0.73 (0.67–0.79)**
≥60	425	163 (38.4)	**0.66 (0.58–0.74)**	**0.67 (0.59–0.77)**
**Occupation**				
Physician	910	551 (60.6)	**1.18 (1.08–1.30)**	**1.18 (1.08–1.30)**
Nurse	2,141	1,217 (56.8)	**1.09 (1.01–1.18)**	**1.09 (1.01–1.18)**
Allied health professional	9,68	472 (48.8)	0.96 (0.87–1.05)	0.96 (0.87–1.05)
Administrative staff	964	451 (46.8)	Ref.	Ref.
Researcher	962	495 (51.5)	1.06 (0.97–1.16)	1.06 (0.97–1.17)
Other	276	131 (47.5)	1.04 (0.91–1.20)	1.05 (0.91–1.20)
**Location of the center**				
Osaka	1,044	563 (53.9)	Ref.	Ref.
Aichi	543	272 (50.1)	0.95 (0.96–1.08)	0.98 (0.96–1.09)
Tokyo	4,634	2,482 (53.6)	1.02 (0.96–1.08)	1.02 (0.96–1.09)

CI, confidence interval; PR, prevalence ratio.
Model 1 was adjusted for age and sex. Model 2 was additionally adjusted for job (physician, nurse, allied health professional, administrative staff, researcher, or others) and location of National Centers (Tokyo, Osaka, or Aichi).

eTable 3. COVID-19 Seroprevalence of each National Center across the fiscal year survey

NCs	2020 fiscal year		2021fiscal year		2022 fiscal year		2023 fiscal year	
	**Participants (N)**	**Seropositive, N (%)**	**Participants (N)**	**Seropositive, N (%)**	**Participants (N)**	**Seropositive, N (%)**	**Participants (N)**	**Seropositive, N (%)**
**Total**	5,224	36 **(0.7)**	5,309	85 **(1.6)**	7,000	1,060 **(15.1)**	6,287	2,974 **(47.3)**
**NCC**	444	7 (1.6)	607	13 (2.1)	522	76 (14.6)	429	176 (41.0)
**NCCHD**	361	6 (1.7)	--	--	1,402	209 (14.9)	1,308	618 (47.2)
**NCGG**	631	3 (0.5)	800	6 (0.7)	806	89 (11.0)	553	245 (44.3)
**JIHS**	2,563	13 (0.5)	2,763	43 (1.6)	2,695	416 (15.4)	2,596	1,250 (48.2)
**NCNP**	657	5 (0.8)	407	4 (1.0)	369	117 (31.7)	316	163 (51.6)
**NCVC**	568	2 (0.3)	732	19 (2.6)	1,206	153 (12.7)	1,085	522 (48.1)

COVID-19, coronavirus disease 2019; JIHS, Japan Institute for Health Security; NC, National Center; NCC, National Cancer Center; NCCHD, National Center for Child Health and Development; NCGG, National Center for Geriatrics and Gerontology; NCNP, National Center of Neurology and Psychiatry; NCVC, National Cerebral and Cardiovascular Center.

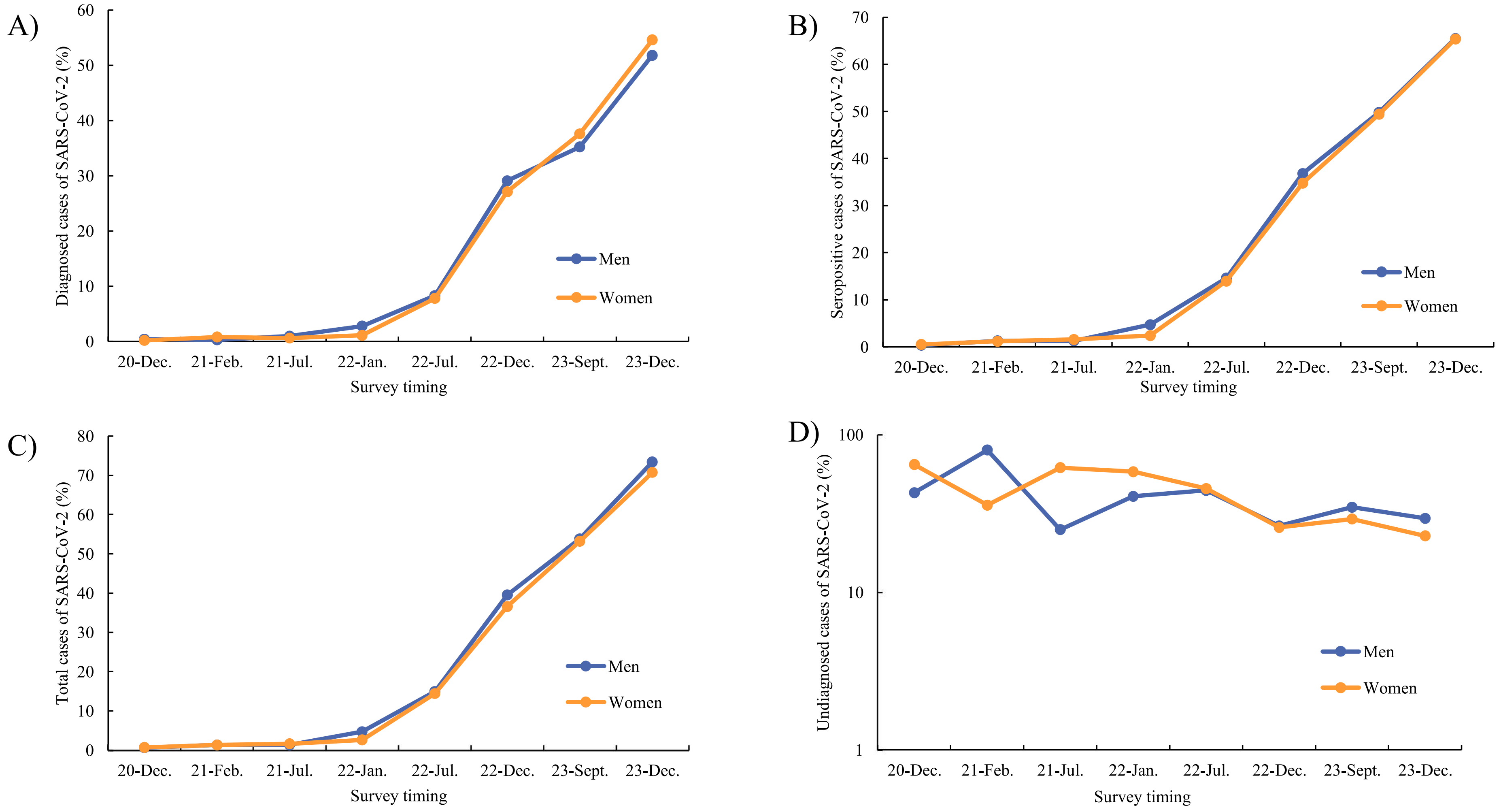

eFigure 1. Trends in SARS-CoV-2 infection among staff at the six national centers in Japan during the pandemic, showing the percentage of diagnosed cases (**A**), seropositive cases (**B**), total cases (**C**), and undiagnosed cases (**D**) by sex

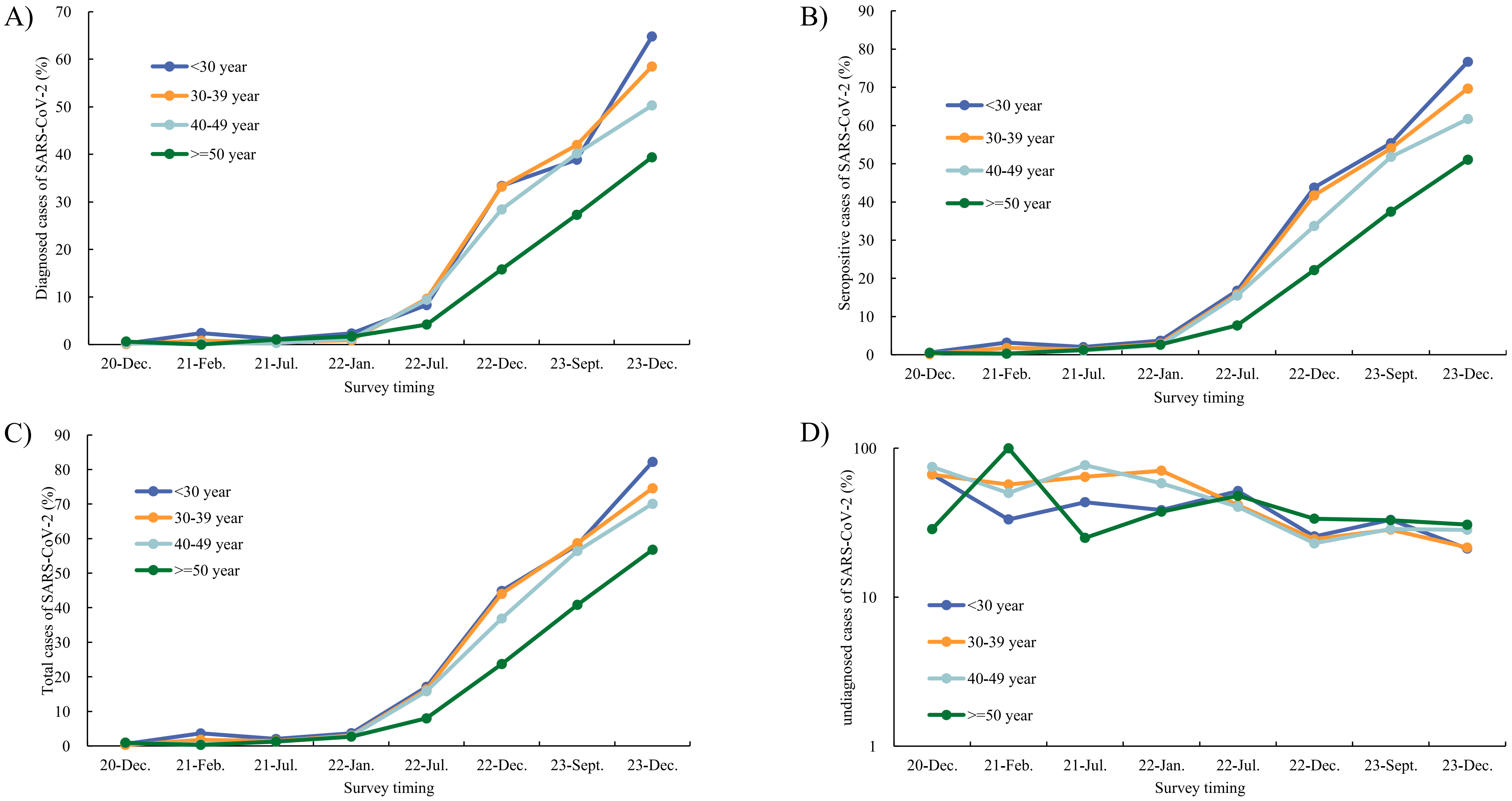

eFigure 2. Trends in SARS-CoV-2 infection among staff at the six national centers in Japan during the pandemic, showing the percentage of diagnosed cases (**A**), seropositive cases (**B**), total cases (**C**), and undiagnosed cases (**D**) by age category

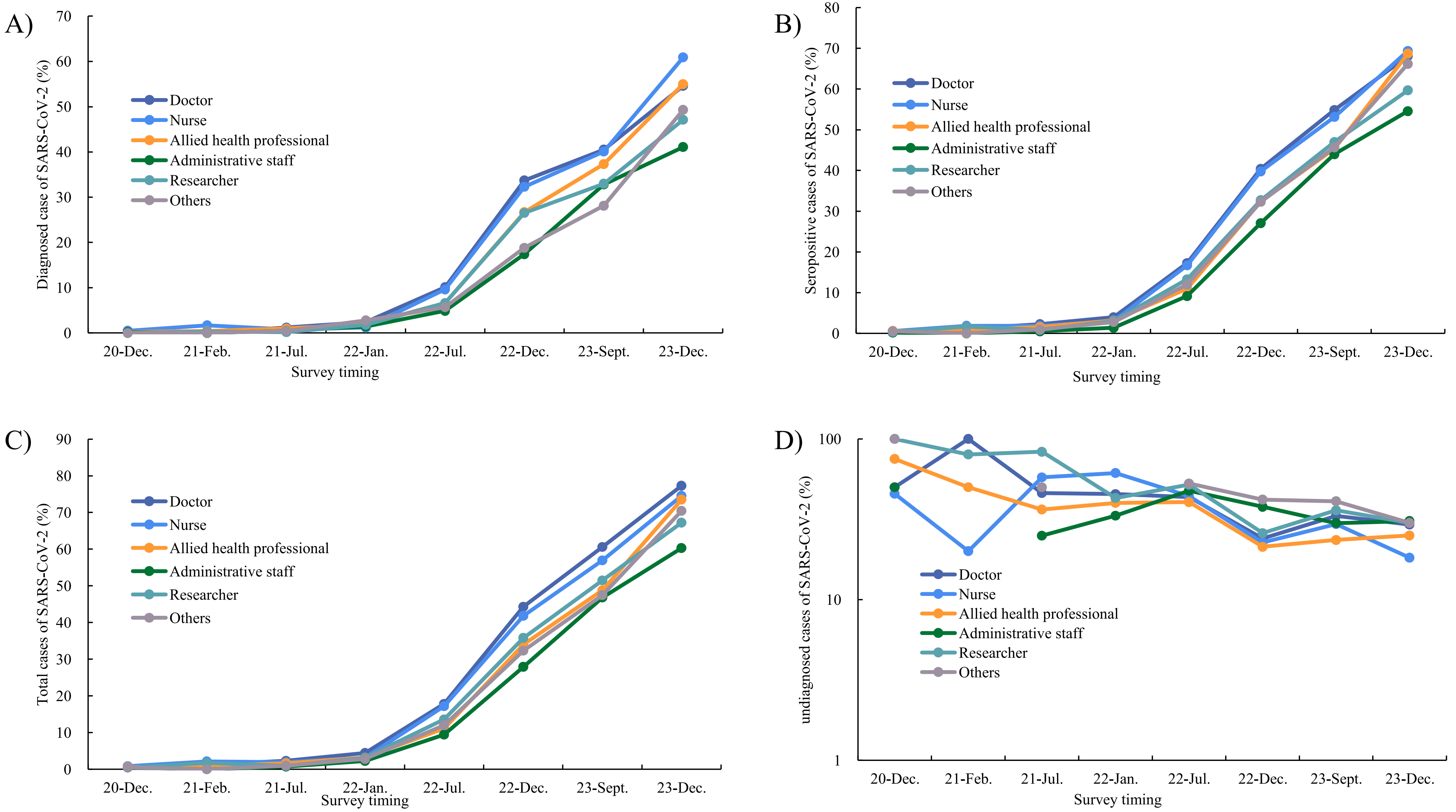

eFigure 3. Trends in SARS-CoV-2 infection among staff at the six national centers in Japan during the pandemic, showing the percentage of diagnosed cases (**A**), seropositive cases (**B**), total cases (**C**), and undiagnosed cases (**D**) by job category

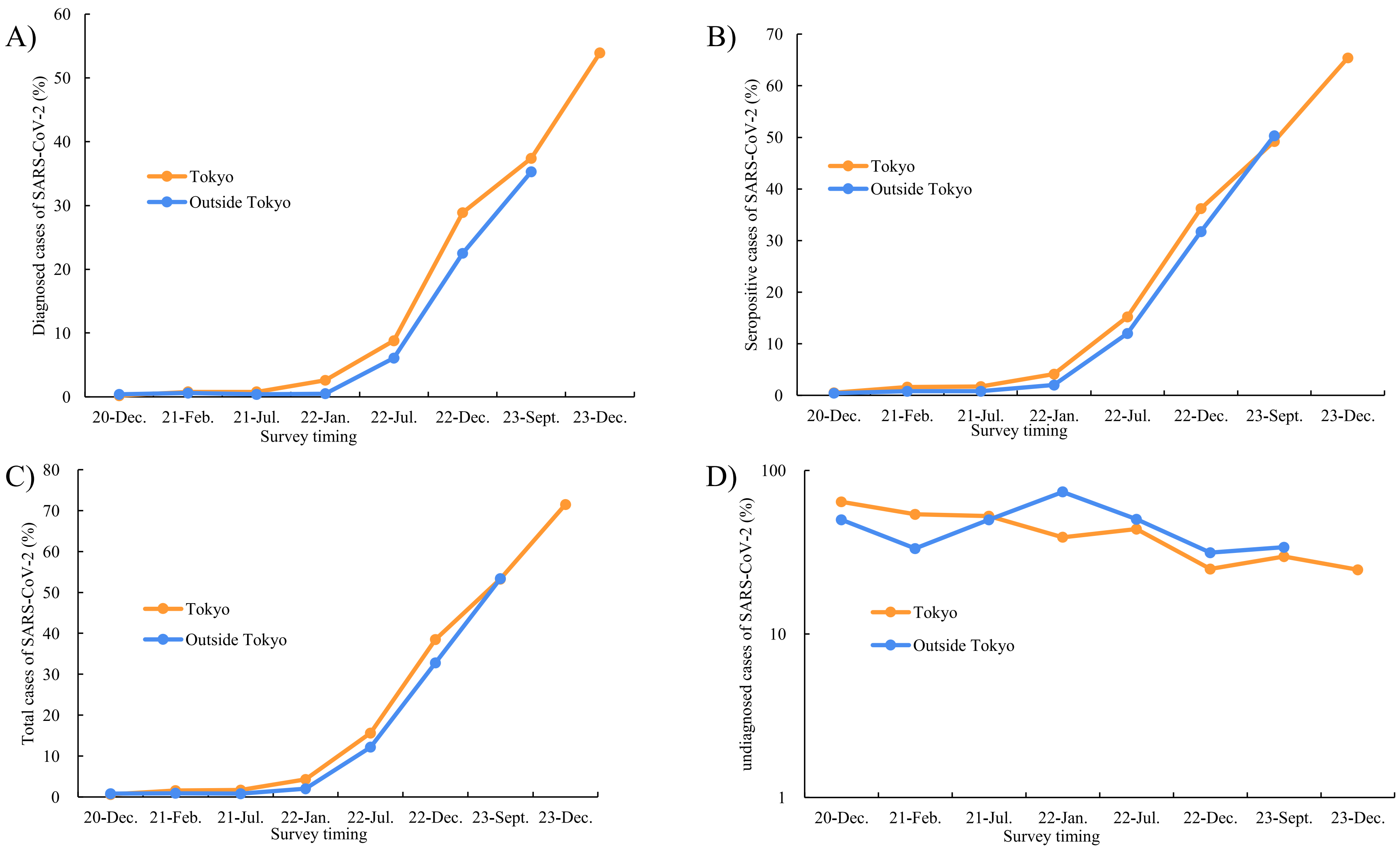

eFigure 4. Trends in SARS-CoV-2 infection among staff at the six national centers in Japan during the pandemic, showing the percentage of diagnosed cases (**A**), seropositive cases (**B**), total cases (**C**), and undiagnosed cases (**D**) by location of national centers
